# Circulating hyaluronan as a marker of endothelial glycocalyx damage in dogs with myxomatous mitral valve disease and dogs in a hypercoagulable state

**DOI:** 10.1016/j.tvjl.2022.105845

**Published:** 2022-07

**Authors:** Sara J. Lawrence-Mills, Melanie J. Hezzell, Sophie E. Adamantos, Iris Chan, Kieran Borgeat, Jessie Rose Payne, Simon Satchell, Gavin I. Welsh, Rebecca R. Foster, Natalie Finch

**Affiliations:** a Bristol Renal, Bristol Medical School, University of Bristol, Bristol, UK; b The Royal Veterinary College, University of London, North Mymms, UK; c Bristol Veterinary School, University of Bristol, Langford, UK; d Langford Vets, Langford House, Langford, UK; e Paragon Veterinary Referrals, Wakefield, UK

**Keywords:** Canine, ELISA, Health monitoring

## Abstract

The endothelial glycocalyx (eGlx) lines the luminal surface of endothelial cells, maintaining vascular health. Glycocalyx damage is pathophysiologically important in many diseases across species however few studies have investigated its breakdown in naturally occurring disease in dogs. The aims of the study were to investigate eGlx damage in dogs with myxomatous mitral valve disease (MMVD) diagnosed on echocardiography, and dogs in a hypercoagulable state diagnosed using thromboelastography (TEG), by measuring serum hyaluronan concentrations. Serum hyaluronan was quantified in dogs with MMVD (*n* = 27), hypercoagulability (*n* = 21), and in healthy controls dogs (*n* = 18). Serum hyaluronan concentrations were measured using a commercially-available ELISA validated for use in dogs. Hyaluronan concentrations were compared among groups using Kruskal-Wallis tests, and post-hoc with Dunn’s tests. Serum hyaluronan concentrations (median [range]) were significantly increased in dogs with MMVD (62.4 [22.8–201] ng/mL; *P* = 0.031) and hypercoagulability (92.40 [16.9–247.6] ng/mL; *P* < 0.001) compared to controls (45.7 [8.7–80.2] ng/mL). Measurement of serum hyaluronan concentration offers a clinically applicable marker of eGlx health and suggests the presence of eGlx damage in dogs with MMVD and dogs in a hypercoagulable state.

## Highlights

• Few studies have investigated endothelial glycocalyx breakdown in disease in dogs.
• Serum hyaluronan is a clinically applicable marker of endothelial glycocalyx health.
• Serum hyaluronan was significantly increased in dogs with mitral valve disease.
• Serum hyaluronan was significantly increased in dogs in a hypercoagulable state.
• Increased serum hyaluronan may indicate glycocalyx shedding in these diseases.

The endothelial glycocalyx (eGlx) is critical in maintaining vascular health, (Alphonsus and Rodseth, 2014) and has been implicated in a plethora of pathophysiological processes in different species (Ueno et al., 2004, Salmon et al., 2012, Kolářová et al., 2014, Lawrence-Mills et al., 2022, In Press). Quantification of eGlx breakdown products such as chondroitin sulfate, hyaluronan, and syndecan-1 in blood is a clinically applicable tool and accepted marker of eGlx health (Broekhuizen et al., 2009, Kubaski et al., 2016) with increases reported in humans with chronic kidney disease (Padberg et al., 2014), sepsis (Nelson et al., 2008), diabetes (Koźma et al., 1996), and decompensated heart failure (Neves et al., 2015). Measurement of hyaluronan as a marker of eGlx damage had been reported in both dogs with septic peritonitis (Shaw et al., 2021) and those receiving fluid boluses (Beiseigel et al., 2021).

Dogs with myxomatous mitral valve disease (MMVD) have vascular endothelial dysfunction, the severity of which increases with disease progression (Jones et al., 2012, Moesgaard et al., 2012). EGlx degradation represents a potential mechanism for endothelial dysfunction (Tarbell and Pahakis, 2006). Cellular studies have demonstrated the abolishment of flow-dependent vasodilation following enzymatic eGlx degradation (Pohl et al., 1991). The eGlx is critical in regulating haemostasis, including harbouring coagulation cascade cofactors (Danielsson et al., 1986, Iba et al., 2019), thus eGlx shedding promotes coagulation. The study aim was to measure serum hyaluronan in dogs as a marker of eGlx damage in MMVD and hypercoagulable states.

Ethical approval for the study provided by the University of Bristol Animal Welfare and Ethics Review Board (Approval number, VIN/16/047; Approval date, 24 November 2016). For the control group, residual serum samples from blood collected for health screening from clinically healthy dogs presented for blood donation to a non-profit organisation (Pet Blood Bank) were used. Health was confirmed in every case by thorough history taking, physical examination, and evaluation of full haematology and serum biochemistry results. For the MMVD group, residual serum samples from dogs presenting to a referral hospital (Langford Vets) with MMVD, were used. Disease severity was classified according to the American College of Veterinary Internal Medicine (ACVIM) consensus statement (Keene et al., 2019). Dogs with congestive heart failure (CHF) were stabilised prior to sample collection. Dogs with co-morbidities were not excluded. Dogs in the MMVD group were not evaluated for concurrent hypercoagulability. For the hypercoagulable group, residual serum samples from dogs in a hypercoagulable state, as defined by thromboelastography (TEG; G > 8 dynes/s), were used. Partnership on Rotational ViscoElastic Test Standardization (PROVETS) guidelines were adhered to (Goggs et al., 2014). These dogs presented with a variety of underlying conditions.

Serum hyaluronan concentrations were determined by a single operator (SLM) using a commercially available sandwich ELISA (Quantikine, R and D Systems, cat number DHYAL0) validated for use in dogs (Beiseigel et al., 2021). The assay was performed according to manufacturer’s instructions. Samples were diluted 1:16 and run in duplicate. A coefficient of variation > 15% was deemed unacceptable. Optical densities were read on a Dynex Opsys MR microplate reader at 450 nm and 570 nm, the latter for correction. Statistical analyses were performed using commercially available software (GraphPad Prism v 9.0, GraphPad Software, Inc.). Data were assessed for normality graphically and by use of the Shapiro-Wilk test. Summary statistics for continuous variables are reported as median (range). Comparisons of categorical variables amongst groups were made using Chi-squared tests and continuous variables using Kruskal-Wallis tests. Post-hoc comparisons between groups were made using the Dunn’s test for multiple comparisons. Type 1 error rate is set at 0.05.

**Fig. 1:**
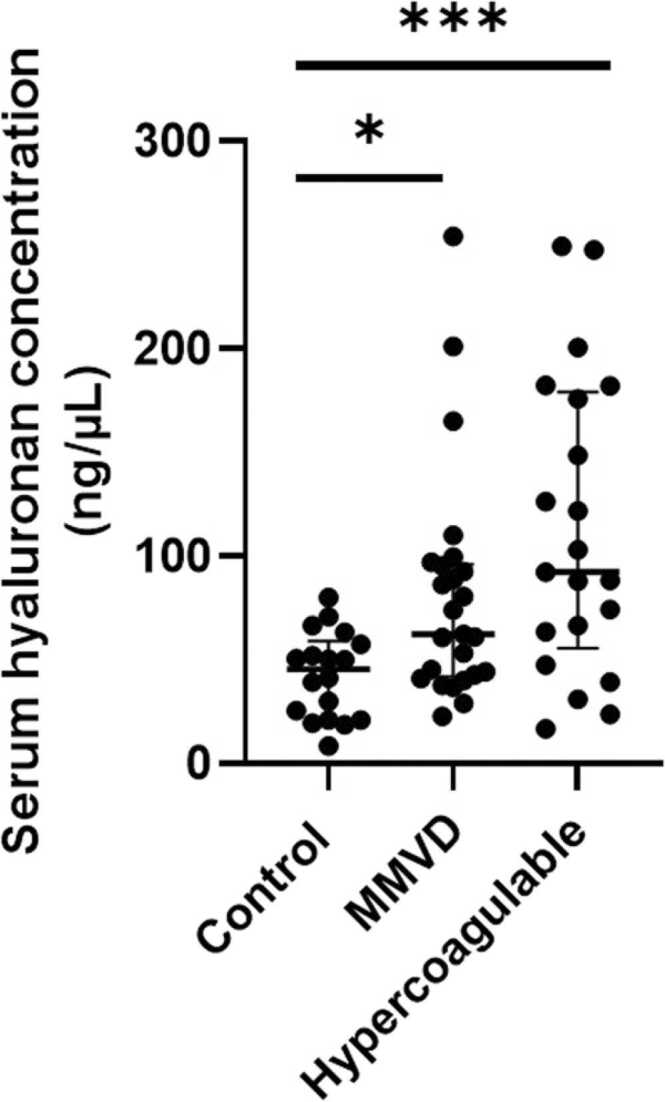
Scatter plot depicting serum hyaluronan concentration in control dogs (*n* = 18) and dogs with different naturally occurring disease states (myxomatous mitral valve disease, MMVD, *n* = 27; and hypercoagulable, *n* = 21). Bars show median and interquartile ranges. * represents *P* < 0.05, * ** represents *P* < 0.001.

**Fig. 2:**
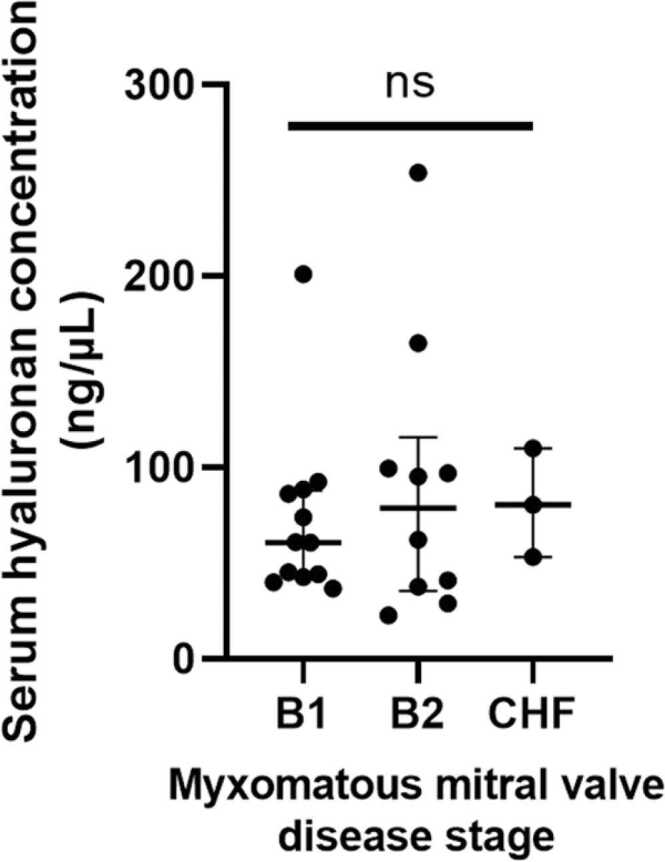
Scatter plot depicting serum hyaluronan concentrations in dogs with different American College of Veterinary Internal Medicine stages of myxomatous mitral valve disease. The congestive heart failure (CHF) group (*n* = 3) comprises dogs in both stage C and D. Bars show median and interquartile ranges. Twelve dogs had stage B1 and ten at stage B2. No significant difference (ns).

Sixty-six dogs were included in the study: 18 control dogs; 27 dogs with MMVD; and 21 dogs in a hypercoagulable state. Population characteristics are presented in Table 1, information about comorbidities and concurrent medication is included in Table 2. The serum hyaluronan concentrations in the control population was 45.7 (8.7–80.2) ng/mL, MMVD population 62.4 (22.8–201) ng/mL, and in dogs in a hypercoagulable state 92.4 (16.9–247.6) ng/mL. Post-hoc pairwise group comparisons revealed differences between control and hypercoagulable (*P* = 0.001) and control and MMVD (*P* = 0.031) groups, but not hypercoagulable and MMVD (*P* = 0.550) groups (Fig. 1). Post hoc pairwise analysis was performed comparing ACVIM stages of MMVD, with stages C and D grouped into a CHF group. No significant differences in serum hyaluronan concentrations were detected between B1 and B2 (*P* > 0.999), B1 and CHF (*P* > 0.999), and B2 and CHF (*P* > 0.999) groups (Fig. 2).

**Table 1:** Comparison of population characteristics for dogs in which serum hyaluronan was measured.

Population	Controls	Myxomatous mitral valve disease	Hypercoagulability*	*P* value
Breed	Pedigree *n* = 18	Pedigree *n* = 25 Crossbreed *n* = 2	Pedigree *n* = 19 Crossbreed *n* = 2	0.894
	Labrador *n =* 6 Golden retriever *n* = 3 Greyhound *n =* 3 Border collie *n =* 1 Dalmatian *n* = 1 German shepherd *n* = 1 Pointer *n* = 1 Poodle *n* = 1 Ridgeback n = 1	Cavalier King Charles spaniel *n* = 11 Border collie *n* = 2 Chihuahua *n* = 2 Springer spaniel *n* = 2 Jack Russell terrier *n* = 1 Labrador retriever *n* = 1 Lhasa apso *n* = 1 Miniature schnauzer *n* = 1 Parson Russell terrier *n* = 1 Shih tzu *n* = 1 Wire-haired fox terrier *n* = 1 Yorkshire terrier *n* = 1	Labrador retriever *n =* 4 Bichon frise *n =* 3 Springer spaniel *n* = 2 Border collie *n =* 1 Bulldog *n =* 1 Cocker spaniel *n =* 1 Dalmatian *n =* 1 English spaniel *n =* 1 Golden retriever *n =* 1 Jack Russel terrier *n =* 1 Lakeland terrier *n =* 1 Pug *n =* 1 West Highland white terrier *n* = 1	
Sex	Female entire *n* = 3 Female neutered *n* = 2 Male entire *n* = 5 Male neutered *n* = 8	Female entire *n* = 1 Female neutered *n* = 15 Male neutered *n* = 11	Female entire *n* = 1 Female neutered *n* = 9 Male entire *n* = 4 Male neutered *n* = 7	0.0055
Age in years Median (range)	6.6 (2.8–10.4)	10.3 (2.1–16.0)	9.0 (2.8–14.3)	< 0.001
Disease classification	Healthy	ACVIM stage** B1 *n* = 14 B2 *n* = 10 C and D *n* = 3	G value (dynes/s)* Median (range) 11.9 (8.3–22.1)	

* as defined by thromboelastography (G >8 dynes/s).

** American College of Veterinary Internal Medicine (ACVIM) stage of myxomatous mitral valve disease.

**Table 2:** Comorbidities and concurrent medication data for clinical populations. Dogs may have multiple comorbidities and be on multiple medications.

Population	Concurrent diseases	Concurrent medications
Hypercoagulable^a^	Hyperadrenocorticism *n* = 4 Idiopathic epilepsy *n* = 4 Immune-mediated haemolytic anaemia *n* = 3 Neoplasia (nasal mass, meningioma, gastrointestinal lymphoma) *n* = 3 Pulmonary thromboembolism *n* = 2 Subarachnoid diverticulum *n* = 2 Aortic thromboembolism *n* = 1 Atopic dermatitis *n* = 1 Endocarditis *n* = 1 Hepatitis *n* = 1 Ischemic myelopathy *n* = 1 Myocarditis *n* = 1 Otitis externa *n* = 1 Protein-losing nephropathy *n* = 1 Pulmonary hypertension *n* = 1 Meningitis *n* = 1 Quadrigeminal cistern arachnoid cyst *n* = 1 Spondylosis *n =* 1 Urinary tract infection *n* = 1 Unspecified hindlimb lameness *n* = 1	Levetiracetam *n* = 4 Amoxicillin-clavulanate *n* = 3 Prednisolone *n* = 3 Maropitant *n* = 2 Phenobarbitone *n* = 2 Potassium bromide *n* = 2 Atenolol *n* = 1 Buprenorphine *n* = 1 Chloramphenicol eye drops *n* = 1 Ciclosporin *n* = 1 Clindamycin *n =* 1 Cyproheptadine *n* = 1 Cytarabine *n* = 1 DDAVP *n* = 1 Dexamethasone *n* = 1 Frusemide *n* = 1 Hydrocortisone *n* = 1 Itraconazole *n* = 1 Lactulose *n* = 1 Meloxicam *n* = 1 Methadone *n* = 2 Methylprednisolone acetate *n* = 1 Pimobendan *n* = 1 Vitamin B12 *n = 1*
Myxomatous mitral valve disease		
B1^b^	Immune-mediated disease *n* = 2 Intervertebral disc disease *n* = 2 Atrial fibrillation *n* = 1 Eosinophilic lymphadenitis *n* = 1 Gastritis *n* = 1 Haematuria *n* = 1 Hepatopathy *n* = 1 Neoplasia (urethral mass) *n* = 1 Otitis externa *n* = 1 Proteinuria *n* = 1 Rhinitis *n* = 1 Syringomyelia *n* = 1 Tracheobronchial collapse *n* = 1	Amoxicillin-clavulanate *n* = 1 Frusemide *n* = 1 Meloxicam *n* = 1 Pimobendan *n* = 1
B2^b^	Intravertebral disc disease *n* = 4 Gastritis *n* = 2 Degenerative joint disease *n* = 1 Haematuria *n =* 1 Hepatopathy *n =* 1 Humeral periostitis n = 1Idiopathic epilepsy *n* = 1 Neoplasia (lymphoma) *n* = 1 Syringomyelia *n* = 1	Maropitant *n* = 2 Benazepril *n* = 1 Buprenorphine *n* = 1 Carprofen *n* = 1 Cefovecin *n* = 1 Frusemide *n* = 2 Pimobendan *n* = 1 Robenacoxib *n* = 1 S-adenosyl methionine *n* = 1 Sucralfate *n* = 1
Congestive heart failure (C or D^b^)	Eosinophilic tonsilitis *n* = 1 Neoplasia (retrobulbar mass) *n* = 1	Benazepril *n* = 2 Frusemide *n* = 2 Pimobendan *n* = 2 Spironolactone *n* = 2 Diphenoxylate hydrochloride *n* = 1

a As defined by thromboelastography (G >8 dynes/s).

b American College of Veterinary Internal Medicine stage of myxomatous mitral valve disease.

This study measured serum hyaluronan as a surrogate marker of eGlx damage in dogs. Increased serum hyaluronan concentrations in dogs with MMVD and in dogs with hypercoagulability is suggestive of eGlx damage in these disease processes. Multiple studies in humans, animal models, and dogs have demonstrated the usefulness of circulating eGlx breakdown product measurement (Williams et al., 2003, Yini et al., 2015, Beiseigel et al., 2021, Shaw et al., 2021). The hyaluronan concentrations identified in the present study control population differ from ranges reported in other studies (Beiseigel et al., 2021). Beiseigel and colleagues reported a baseline median hyaluronan concentration of 17.4 ng/mL with an inter-quartile range (IQR) of 37.3 ng/mL in one group and 25.6 ng/mL; IQR, 25.6 ng/mL in another. Differences in the control populations; including differences in age, breed, and sex distribution, as well as different presentation of the data, precludes direct comparison between studies.

We postulate that dogs in a hypercoagulable state have increased circulating serum hyaluronan due to eGlx shedding. The eGlx is a known reservoir for enzymatic cofactors involved in the coagulation cascade (Ince et al., 2016, Sieve et al., 2018). EGlx shedding may develop as a result of ‘glycocalyx-degradation factors’ such as reactive oxygen species, matrix metalloproteinases and heparinases released in response to inflammation (Sieve et al., 2018).

Dogs with MMVD have known endothelial dysfunction (Puglia et al., 2006, Jones et al., 2012, Moesgaard et al., 2012), increased circulating serum hyaluronan suggests eGlx damage may be associated with this. Atrial and B-type natriuretic peptides (ANP and BNP) cause eGlx shedding in rodent models and human patients (Bruegger et al., 2011, Jacob et al., 2013). Both ANP and BNP are elevated in dogs with MMVD (Tarnow et al., 2009), natriuretic peptide-mediated eGlx damage is therefore plausible. Although neither marker was measured directly in the present study, ACVIM disease stage can be considered a surrogate marker for these neurohormones (Ogawa et al., 2021).

The diagnostic and prognostic potential of eGlx study is increasingly demonstrated in human patients (Dane et al., 2014, Padberg et al., 2014, Ikeda et al., 2018). Significant further research is required to explore its application in eGlx health monitoring in dogs. Limitations of the present study include the measurement of a single eGlx component, potential for non-endothelial sources of hyaluronan, and a small sample size increasing the risk of type 2 statistical error. This is particularly pertinent for the comparisons between ACVIM MMVD disease stages. A further confounding factor was the presence of comorbidities that may influence eGlx health. In addition, the eGlx may be influenced by age and sex; eGlx thickness decreases in advanced age in humans and rats (Salmon et al., 2012, Machin et al., 2018), coroborated by the finding that serum hyaluronan increases with age in humans (Lindqvist and Laurent, 1992) as well as reported sex-linked differences in eGlx health in humans (Brands et al., 2020). Increased age in the diseased groups may have contributed to the significantly higher hyaluronan concentrations in this study. Future research should corroborate findings with multiple measurements of eGlx health (Schmidt et al., 2016) as well as direct visualisation measurements.

This study identified increased serum hyaluronan concentrations in dogs with MMVD and hypercoagulability suggesting eGlx damage in these disease states, demonstrating the potential of this marker for studying the eGlx in dogs.

## Declaration of Competing Interest

MSD provided a financial award to Sara Lawrence-Mills (nee Hillyer) to support her presentation of the research at the British Small Animal Veterinary Association Congress 2018 and the Southern European Veterinary Conference 2018. None of the authors has any financial or personal relationships that could inappropriately influence or bias the content of the paper.
